# Neurally adjusted non-invasive ventilation in patients with chronic obstructive pulmonary disease: does patient–ventilator synchrony matter?

**DOI:** 10.1186/s13054-014-0670-2

**Published:** 2014-11-28

**Authors:** Stefano Nava, Lara Pisani

**Affiliations:** Respiratory and Critical Care, Sant’Orsola Malpighi Hospital, via Massarenti 9, Bologna, Italy

## Abstract

Patient–ventilator interaction represents an important clinical challenge during non-invasive ventilation (NIV). Doorduin and colleagues’ study shows that non-invasive neurally adjusted ventilatory assist (NAVA) improves patient–ventilator interaction compared with pressure support ventilation in patients with chronic obstructive pulmonary disease. There is no doubt nowadays that NAVA is the most effective mode of improving the synchrony between patient and machine, but the key question for the clinicians is whether or not this will make a difference to the patient’s outcome. The results of the study still do not clarify this issue because of the very low clinically important dyssynchrony, like wasted efforts, in the population studied. Air leaks play an important role in determining patient–ventilator interaction and therefore NIV success or failure. Apart from the use of a dedicated NIV ventilator or specific modes of ventilation like NAVA, the clinicians should be aware that the choice of interface, the humidification system and the appropriate sedation are key factors in improving patient–ventilator synchrony.

A lot of emphasis has been placed recently on the problem of patient–ventilator interaction during non-invasive ventilation (NIV). This issue is particularly important in patients with chronic obstructive pulmonary disease during an episode of acute hypercapnic respiratory failure.

In their elegant study, Doorduin and colleagues show that the use of non-invasive neurally adjusted ventilatory assist (NAVA) improved patient–ventilator synchrony compared with pressure support ventilation (PSV), delivered either by a dedicated NIV platform or by an ICU ventilator with dedicated software [1]. Indeed, they demonstrated that automated analysis of ventilator pressure and diaphragm electrical activity waveforms allowed an objective detection of patient–ventilator interaction.

This study largely confirmed the results already described in other investigations performed in heterogeneous groups of critically ill patients [2,3]. There is no doubt nowadays that NAVA is the most effective mode of improving the synchrony between patient and machine, but the key question for the clinician is whether or not this will make a difference to the patient’s outcome.

In other words, does patient–ventilator synchrony matter? In invasively ventilated patients, a high incidence of asynchrony is associated with a prolonged duration of mechanical ventilation and a higher rate of tracheotomy during assisted mechanical ventilation [4]. This association was mainly due to a nonappropriate setting of the ventilator parameters (that is, a high inspiratory pressure or a less sensitive trigger), rather than the patient’s clinical severity or ventilatory modes [5]. A higher discomfort in patients receiving NIV for acute and even chronic respiratory failure was reported, but apparently did not influence gas exchange or any other clinical parameter [6]. Comfort is a main goal to achieve during NIV since it may determine the tolerance of the patient, which is still one of the main causes of NIV failure and therefore of intubation [7]. NIV is a semi-open system and air leaks around the mask are very likely to occur, particularly in the first few hours of ventilation when the patient needs to adapt to this non-natural breathing. Air leaks are the major cause of poor synchrony during NIV [6] and therefore dedicated NIV platforms and ICU ventilators using a specific module have been developed to minimize this problem. *In vivo* and bench assessment showed that these ventilators, particularly the former, are able to almost avoid the occurrence of mismatching [8]. In contrast, the study by Doorduin and colleagues showed a considerable amount of dyssynchrony especially using the NIV platform during PSV [1].

The authors have used a sophisticated automatic algorithm to define acceptable synchrony (that is, an error between electrical activity of the diaphragm and airway pressure above 20%) [1].

The threshold of this definition was arbitrarily chosen. It was not clear what could be the clinical impact of this discrepancy, if not that this threshold value is associated with a higher occurrence of wasted efforts. Surprisingly the number of these events, the only ones associated with the worst outcomes in intubated patients [4], was extremely low in the three different trials. On one hand this may be explained by the fact that PSV and NAVA are, besides small differences, equally effective in avoiding major asynchrony events, but on the other that they are related by the nature of Doorduin and colleagues’ study [1]. As a matter of fact the patients enrolled in the study were recovering from an episode of acute respiratory failure with a normal pH, and were therefore ventilated with a low inspiratory pressure (mean 7 cmH_2_O) that is very unlikely to induce the phenomenon of wasted efforts.

In our view the most striking difference highlighted by the study was the huge discrepancy in the time to trigger the ventilator between NAVA and PSV, which has usually been explained by the fact that the ventilator with NAVA is triggered directly by electrical activity of the diaphragm, regardless of the presence of intrinsic positive end-expiratory pressure (PEEP). The mean level of set PEEP during PSV was around 6 cmH_2_O, and therefore was close enough to balance the level of intrinsic PEEP recorded during an acute exacerbation of chronic obstructive pulmonary disease patients [9]. The presence of air leaks was the major driver of the delay rather than the presence of intrinsic PEEP.

When the respiratory drive is elevated, such as in the case of acute respiratory failure, the scenario may be different. A mathematical model has shown that, in the presence of an inspiratory leak proximal to the airway, opening can be accompanied by marked variations in duration of the inspiratory phase and in autoPEEP [10], and this may be a rationale for using NAVA as a preferred method of NIV.

Despite the fact that the clinical impact of a poor patient–ventilator interaction is still not clear, the role of the mode of ventilation still needs to be elucidated, while the presence of air leaks should be always minimized.

Other than using a ventilator specifically compensating for leaks, the clinicians should be aware that the choice of the interface, the humidification system and, last but not least, the appropriate sedation have been shown to improve the patient’s tolerance of NIV [11].

## Abbreviations

NAVA: Neurally adjusted ventilatory assist
NIV: Non-invasive ventilation
PEEP: Positive end-expiratory pressure
PSV: Pressure support ventilation
